# Tissue Engineering a Biological Repair Strategy for Lumbar Disc Herniation

**DOI:** 10.1089/biores.2015.0034

**Published:** 2015-11-01

**Authors:** Grace D. O'Connell, J. Kent Leach, Eric O. Klineberg

**Affiliations:** ^1^ Department of Mechanical Engineering, University of California, Berkeley, Berkeley, California.; ^2^ Department of Biomedical Engineering, University of California, Davis, Davis, California.; ^3^ Department of Orthopedic Surgery, University of California, Davis Medical Center, Davis, California.

**Keywords:** biomaterials, disc degeneration, disc mechanics, intervertebral disc, low back pain, regenerative medicine

## Abstract

The intervertebral disc is a critical part of the intersegmental soft tissue of the spinal column, providing flexibility and mobility, while absorbing large complex loads. Spinal disease, including disc herniation and degeneration, may be a significant contributor to low back pain. Clinically, disc herniations are treated with both nonoperative and operative methods. Operative treatment for disc herniation includes removal of the herniated material when neural compression occurs. While this strategy may have short-term advantages over nonoperative methods, the remaining disc material is not addressed and surgery for mild degeneration may have limited long-term advantage over nonoperative methods. Furthermore, disc herniation and surgery significantly alter the mechanical function of the disc joint, which may contribute to progression of degeneration in surrounding tissues. We reviewed recent advances in tissue engineering and regenerative medicine strategies that may have a significant impact on disc herniation repair. Our review on tissue engineering strategies focuses on cell-based and inductive methods, each commonly combined with material-based approaches. An ideal clinically relevant biological repair strategy will significantly reduce pain and repair and restore flexibility and motion of the spine.

## Introduction

**FIG. 1.**
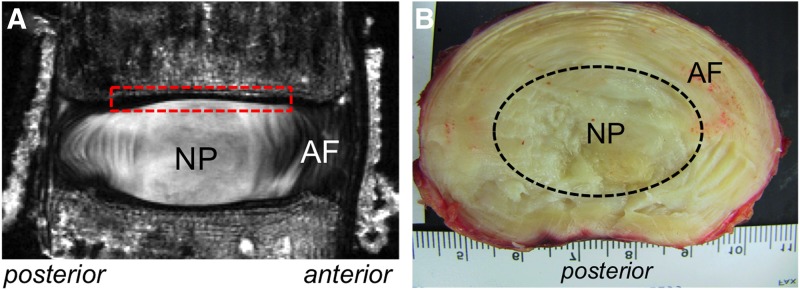
**(A)** High-resolution magnetic resonance image (MRI) from the midsagittal slice of a nondegenerated lumbar intervertebral disc. Red dashed box represents a region covered by the cartilaginous end plate, which is located on the superior and inferior end of the disc. **(B)** Cross-sectional view of healthy nondegenerated lumbar disc. The approximated nucleus pulposus (NP) region is outlined by the black dashed oval. Scale in background represents 1 mm increments. The annulus fibrosus (AF) structure can be identified on both images.

The human lumbar intervertebral disc (IVD) comprises three distinct components, including a nucleus pulposus (NP) surrounded by a lamellar annulus fibrosus (AF), both of which are sandwiched between the cartilaginous end plates on the vertebral bodies (Fig. 1).^1^ The three subcomponents comprise mostly water, proteoglycans, and collagen (Table 1).^2–5^ The AF is populated with fibroblast-like cells and stem cells.^6–9^ The AF collagen composition in the outer region comprises mostly collagen type I, which decreases toward the NP, while the collagen type II content increases from the outer to the inner AF (Table 1).^10^ The NP is derived from notochordal cells that either disappear or are replaced by chondrocyte-like NP cells during development.^11^ However, NP cells retain some notochordal molecular markers, which have increased interest in defining the cell phenotype (see Ref.^12^ for an in-depth analysis of work and challenges in the area). Extracellular matrix produced by NP cells comprises predominantly negatively charged proteoglycans and randomly aligned collagen type II fibers.^13–15^ Age-related changes are characterized by cellular apoptosis, a decrease in collagen and proteoglycan content that leads to water loss.^2,4^ These changes may lead to weakening of the AF, allowing the NP to bulge and potentially herniate through an annular fissure, causing neural compression and clinical symptoms (i.e., disc herniation).

**Table 1.** **Range of Reported Biochemical Compositions in the NP and Inner and Outer AF of ND and Degenerate Discs**

	Water	GAG (%/WW)	GAG (%/DW)	Col. (%/WW)	Col. (%/DW)	% Type II Col.
NP						
ND	70–82	9–15	45–55	5–6	18–23	80–100
D	70–75	5–9	15–25	6–8	20–27	
Inner AF						
ND	75–80	9–14	40–47	18–20	55–60	50–70
D	73–75	6–9	17–25	10–13	30–55	55–75
Outer AF						
ND	65–72	5–8	10–18	40–45	80–85	0–25
D	65–70	3–8	8–10	24–26	60–75	10–30

Data were compiled from values reported for human discs in Refs.^2–5^

AF, annulus fibrosus; Col. Collagen; D, degenerate; DW, dry weight; GAG, glycosaminoglycan; ND, nondegenerate; NP, nucleus pulposus; WW, wet weight.

Clinical issues causing back pain are the second leading cause for disability in Americans, accounting for 17% of disabled persons.^16^ The origin for low back pain can be difficult to diagnose, making long-term treatment with improved clinical outcomes a significant challenge for surgeons and bioengineers. Two of the most common spinal issues associated with low back pain include disc herniation and degeneration. Understanding the root cause of pain from degeneration is difficult due to a high prevalence of asymptomatic individuals with disc degeneration.^17^ In contrast, pain caused by a herniated disc may be easier to identify as the protruding material impinges on spinal nerves, resulting in low back pain and/or sciatica (i.e., radiating leg pain).

The purpose of this review is to identify a critical need for biological repair strategies for disc herniation treatment. Future repair strategies can be greatly improved using recent advances in tissue engineering and regenerative medicine techniques. Designing repair or replacement strategies that better mimic the natural function of the healthy disc requires an understanding of the disc's structure–function relationship. Therefore, this review evaluates the current knowledge in human IVD biomechanics and recent work that has applied tissue engineering techniques for disc repair.

## Clinical Disc Herniation

Lumbar disc herniation (LDH) is one of the most common clinical diagnoses seen in spinal practice.^18^ Notable risk factors for disc herniation include manual labor, prolonged driving, and patients who work in positions of sustained lumbar flexion or rotation.^19–21^ Over 3 million people in the United States (1–2% of the population) have a herniated lumbar disc with associated symptoms, including lower back pain and sciatica.^22^

**FIG. 2.**
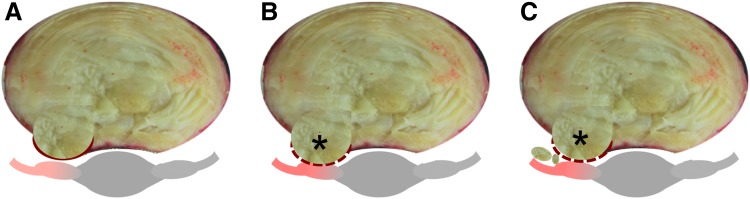
Schematic of three types of disc herniations through the posterior-lateral AF, which is the most common location for disc herniations. **(A)** Disc protrusion of nuclear material through the intact AF. **(B, C)** Damage to the AF (dashed line) allows NP material to extrude from the disc (i.e., noncontained herniation; asterisks). **(C)** Represents sequestration of nuclear material, where NP material becomes loose from the disc space and may further impinge on spinal nerves (red highlights on gray nerves).

**FIG. 3.**
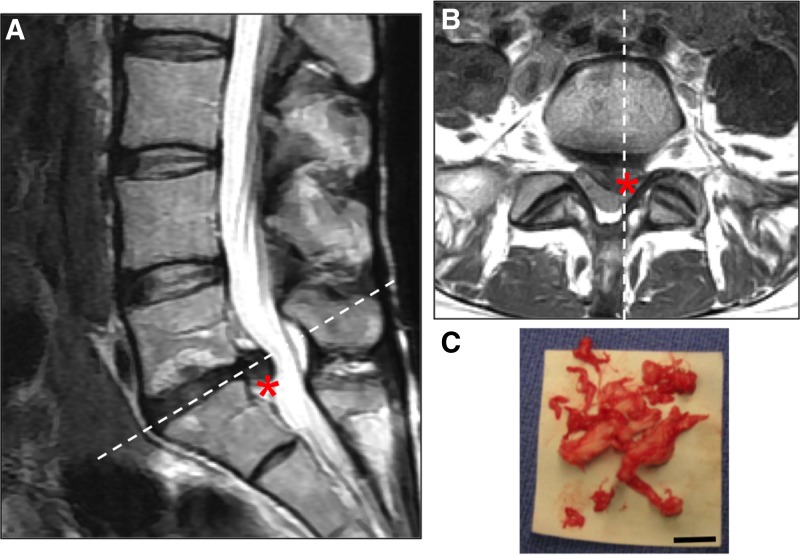
**(A)** Midsagittal and **(B)** axial sections of T2-weighted MRI from a 42-year-old female with lumbar radiculopathy. The white dashed lines indicate the plane of orientation in the axial and midsagittal views, respectively. The patient had a left-sided paracentral disc herniation with compression of the traversing S1 nerve root (represented by *). **(C)** Photograph of disc material successfully removed during a surgical procedure to treat painful disc herniation. Scale bar represents 20 mm.

Disc herniation is defined as localized NP material that protrudes beyond the margins of the disc space. The disc can exhibit different degrees of herniation from disc bulging to an extruded disc where NP material exits the disc space area (Fig. 2).^23^ The least severe condition is a protrusion of disc material where the herniation causes mild compression on the spinal nerves, but the disc material is contained within the disc space (Fig. 2A). A noncontained herniation is one in which the protruding NP material is no longer restrained by the AF (Fig. 2, dashed line). Noncontained herniations are classified as being either extruded or sequestered (Fig. 2B, C, respectively) and can be clearly visualized on magnetic resonance image (MRI; Fig. 3). Noncontained herniations can lead to both chemical and mechanical nerve compression, resulting in neurologic dysfunction, including pain, sensory deficits, and weakness in the lower back and leg (Fig. 2, gray tissue with red highlights in schematic).^24^

### Nonoperative treatment

Nonoperative management remains the foundation of initial treatment for the majority of adult patients with LDH.^22,25^ Approximately 80% of patients achieve a good recovery from disc herniation using nonoperative treatments.^26–29^ One potential reason for high successful outcomes is that the herniated nucleus pulpous often resorbs, which has been demonstrated through diagnostic imaging of tissue in the spinal canal.^30–34^ Even when the herniated tissue occupies 55–80% of the canal diameter (average = 66%), Cribb et al. reported that the herniated tissue volume decreased by 80% within 2 years without significant neurologic deterioration (14/15 patients).^32^ These findings, however, may be limited due to the small sample size. Furthermore, longitudinal studies have reported that up to 30% of patients continue to experience low back pain, inhibiting ∼20% of patients from returning to work, which can have significant psychological and economical effects.^35^ Therefore, an effective repair strategy would provide long-term pain relief and restore spinal function.

### Surgical treatment

Over a million surgical discectomy procedures occur in the United States each year to treat painful disc herniations,^36^ and discectomy remains an important treatment option for patients who experience persistent pain following nonoperative treatment. Discectomy treatment involves removal of up to 2 g of NP material, altering disc mechanics, load distribution, and may increase the rate of disc degeneration.^37–39^ However, discectomy treatment may have a more profound benefit in early pain management for herniation, with significant improvement in outcome measures 1 year postsurgery.^22,26,40^ Unfortunately, the advantage for surgery fades over time, with no significant differences between pain outcome measures and return-to-work rates between operative and nonoperative strategies after 4 years.^18,26,41,42^ These studies suggest that while discectomy has significant short-term gains, patients who can be managed nonoperatively may have similar long-term outcomes to patients treated operatively.

Importantly, disc herniation and discectomy reduce NP material and cause annular damage. These changes significantly modify the tissue structure and load sharing between spinal components, including increased loading applied to the AF, adjacent discs, and facet joints.^38,43,44^ Recent work in developing a biological repair strategy for the IVD has focused on engineered NP tissues using cell-based approaches. Therefore, the ideal repair tissue should restore the biomechanical function of a healthy functional disc joint, which is the focus of the following section.

## IVD Mechanical Properties

The primary function of the IVD is to absorb and distribute large loads placed on the spine during daily activities. The IVD joint allows for six degrees of rotation and displacement. Activities of daily living place large mechanical demands on the lumbar spine, including repetitive combined loading in bending, torsion, and compression.^45–47^ Primarily, the disc is loaded under axial compression due to gravitational and muscular forces. Bending, torsion, and lateral bending are also important loading conditions experienced by the disc during daily living. Understanding disc mechanobiology with injury and degeneration will be important for developing a biological repair strategy that mimics the healthy disc and its subcomponents.

Mechanical testing of NP or AF explants presents significant challenges due to altered boundary conditions, resulting in excessive NP swelling or limited AF fiber engagement during uniaxial tension.^47–50^ The negatively charged proteoglycans in the NP are crucial to the disc's recovery behavior during bed rest by attracting water molecules into the tissue, thereby increasing internal pressure and disc height.^51–53^ *In vitro* testing of the NP under unconfined compression suggests low mechanical stiffness (Young's modulus ∼5 kPa).^54,55^ However, *in situ* boundary conditions act to increase the internal pressure, causing axial stresses to be distributed radially through the NP to the AF.^38,56–58^ Moreover, stresses are transferred from the disc to surrounding tissues, including the vertebral bodies, facet joints, and surrounding musculature. Therefore, to understand the IVD mechanical function, facet joints are often removed for testing, and that model is the focus of the findings reported here.

### Axial compression

The nonlinear poroelastic behavior of the bone–disc–bone motion segment under compression has been the focus of extensive examination. Axial compression decreases disc height and increases intradiscal pressure.^59,60^ The NP is thought to be critical in supporting the disc at low stresses, and then loads are transferred radially to the AF, resulting in the AF directly supporting axial compressive loads at higher stresses.^38,39,43,53,61^ Compressive loads are also directly supported by the annulus through circumferential hoop tension.^62–64^ The compressive Young's modulus, a measure of the disc's material properties, of healthy nondegenerated discs ranges from 5 to 20 MPa and decreases with degeneration.^38,65^ Static axial compression under physiological levels (∼1 MPa stress) has demonstrated disc strains up to 15%, with degeneration resulting in larger disc strains partially due to a lower disc height.^3,38,57,65–69^

The disc height decreases with age and degeneration, increasing axial strains and load distribution toward the facets.^38,44^ The decrease in disc joint compressive mechanics closely mirrors changes observed for NP explants with degeneration,^55^ supporting the notion that the NP is crucial for absorbing and transferring disc joint loads under moderate levels of physiological compression. However, it is important to note that there are relatively few experimental studies that have reported the effect of degeneration on disc and tissue mechanics^38,70,71^ due to complete disc collapse in severely degenerated discs and the limited use of grading schemes until the 1990s.^72,73^

**FIG. 4.**
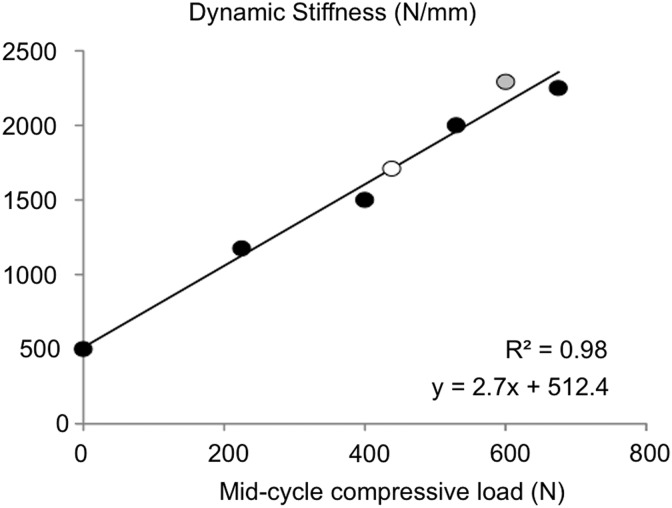
Dynamic stiffness plotted with respect to the mid-cycle axial compression load demonstrates a strong linear relationship. Data were compiled from reported values from Refs.^3,64,74^ (black, white, and gray).

Functional mechanical properties, measured under dynamic compression, are dependent on preload and loading rate, making comparison of mechanical properties across studies challenging. Based on multiple reports in the literature, dynamic stiffness is strongly correlated with the axial compression preload (e.g., mid-cycle compressive load; Fig. 4).^3,64,74^ Table 2 provides a summary of human disc mechanical properties, with properties separated for the effects of degeneration (nondegenerate [ND] vs. degenerate [D]) and discectomy.

**Table 2.** **Mechanical Properties of Intact and Discectomy Intervertebral Discs Under Axial Compression, Axial Tension, Bending (i.e., Flexion, Extension, and Lateral Bending), Axial Torsion, and Shear**

	Intact	Discectomy
Compressive modulus (MPa)		
ND	10–20	7–14
D	5–12	8
—	4–25	2–29
Tension modulus (MPa)		
ND	2.6–3.5	
—	n/a	2.5
Bending stiffness (N × mm/deg)		
Flexion	500–2200	
Extension	1100–2500	
Lateral	1800–3800	
Torsional stiffness (N × mm/deg)		
ND	700–1100	200–1000
D	600–1800	500–1400
Shear stiffness (N/mm)		
Lateral	40–300	200–265
Anterior–posterior	20–300	165–500

Values for ND and D discs are separated when noted in the respective studies. “—” Represents pooled data for ND and D discs reported. Values were compiled from data reported in Refs.^3,38,57,66–71,91,151^

### Bending and torsion

The natural curvature of the IVD provides some inherent degree of bending at each disc level. Furthermore, the collagen fiber orientation in the AF suggests that the tissue provides strong tensile support in axial rotation or torsion. However, experimental methods used to apply a moment arm can vary widely across studies, creating differences in the axis of rotation, which accounts for some variability in value reported in the literature.^38,75,76^ For example, flexion or extension can be applied by using an offset compressive load or a follower load applied about the disc centroid.^38,43,75,76^

**FIG. 5.**
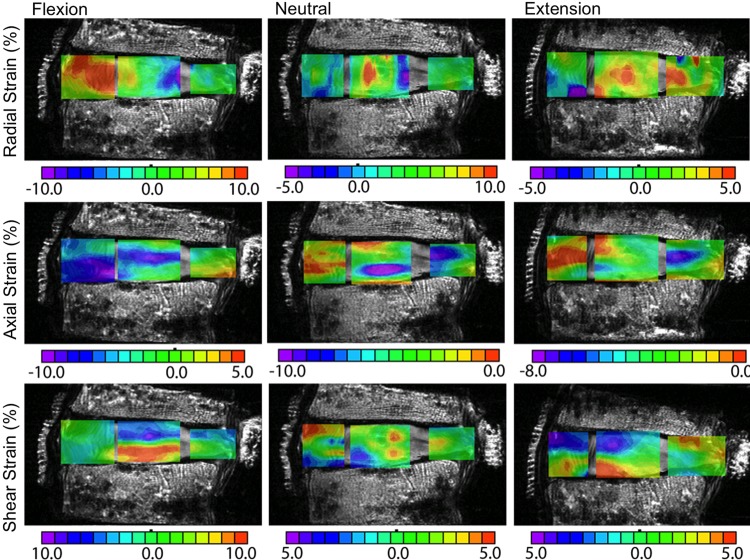
Representative strain maps of the same nondegenerate samples under flexion (first column), neutral (second column), and extension (third column) following discectomy. Radial strains are shown in the first row, axial strains in the second row, and shear strains in the third row. Note that the 0% strain position changes for each strain component. Peak strain locations were similar following discectomy; however, the peak strains were greater following discectomy. Figure adapted from data reported in O'Connell et al.^38^

Based on MRI during flexion and extension, individual discs experience up to 8° of bending pre-disc level *in situ*.^77,78^ Internal disc mechanics under flexion and extension reveal significant stress distribution between the anterior and posterior AF (Fig. 5, first and third columns).^38,66,79^ More specifically, the healthy NP migrates posteriorly during flexion and anteriorly during extension, increasing stresses applied to the AF.^46,80–82^ The load distribution under bending and compression results in high tensile strains in the axial, radial, and circumferential directions (5–10%) due to the Poisson's ratio for NP and AF material being greater than 0.5 (NP: ∼0.6; AF: 0.6–2.1).^49,54^

Interestingly, physiological levels of flexion and extension can cause radial tensile strains in the AF, which suggests some amount of separation between the fibril layers.^38,57,61,83^ That is, in flexion, the anterior AF experiences tensile strains, while the posterior AF experiences tensile strains under extension (Fig. 5, first and third columns, first row).^38^ The decrease in water content and intradiscal pressure with degeneration and discectomy results in even greater tensile radial strains in the AF that may lead to annular tears or microfractures, which frequently originate at the NP-AF boundary.^38,57,83,84^

Torsional loading from axial rotation is an important loading modality to understand the disc's function during daily activities, especially in combination with axial compression or bending.^85–87^ Torsional strength of the disc joint is provided in equal parts by vertebral bodies and the IVD.^88^ Axial rotation of individual discs ranges from 0° to 5° under physiological loading conditions and increases throughout the lumbar spine (i.e., L1/L2 vs. L4/L5).^88,89^ Collagen fibers in the AF are oriented at ±30°–45° with respect to the horizontal plane, providing the disc with a unique ability to withstand large rotational deformations before failure (>10° of rotation at failure).^90^ In contrast to the highly nonlinear behavior observed under compression and bending, torque in the disc increases linearly with rotation,^90,91^ which may be due to preloading the collagen fibers during axial compression preload. However, the coupled compression–torsion mechanical response is not well understood.

Haughton et al. reported an increase in axial rotation in patients with abnormal or painful discs.^89^ Recent work by Bisschop et al. demonstrated an increase in torsional stiffness with degeneration.^70^ Findings from these studies suggest that degenerated or injured discs are likely to experience more rotation and higher torque, which may contribute to increased load transferred to surrounding tissues and warrant further research.

### Effect of treatment on mechanics

Invasive treatment for painful herniation includes removing NP material and additional damage to the posterior-lateral AF,^37^ altering the disc's composition and mechanical properties. In general, removing NP material, increases disc strains and decreases the Young's modulus in tension and compression (Table 2).^71^ Significant changes in compressive mechanical function may advance the degenerative cascade in the affected joint and surrounding tissues or disc levels.

**FIG. 6.**
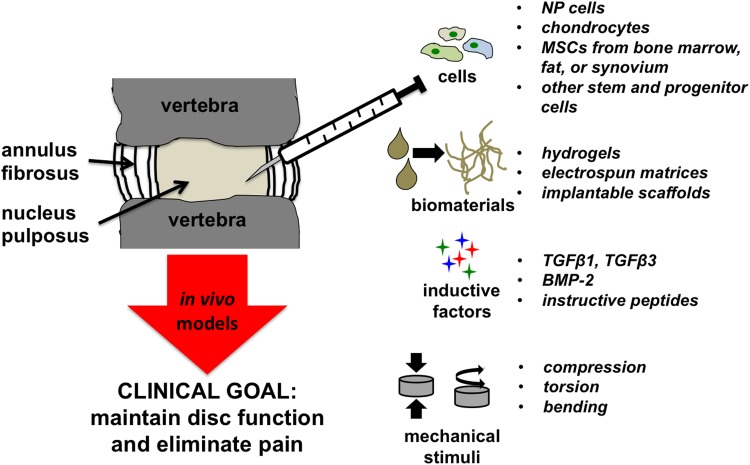
Schematic demonstrating the most common factors applied in tissue engineering approaches for treating herniated and degenerated discs.

Recent clinical strategies for spine repair have moved toward maintaining disc joint mechanics through total disc arthroplasty using metal and plastic components, which have a limited life span in the body.^92–94^ More recently, research has focused on using tissue engineering or regenerative medicine strategies to develop biological repair strategies for injured or degenerated discs.^95^ Successful application of biological repair strategies will need to recapitulate the biochemical composition and act to distribute and absorb large complex loads similar to the healthy native joint. Tissue engineering approaches under investigation to regenerate damaged NP tissue toward healthy tissue, including cell- and material-based approaches, are described in the following sections (Fig. 6).

## Tissue Engineering Approaches to Repair or Replace Disc Tissue

### Cell-based strategies to disc repair

The IVD is poorly cellularized with an average cell density of 4 × 10^3^ cells/mm^3^ in the NP and 9 × 10^3^ cells/mm^3^ in the AF, decreasing significantly with age.^96^ The NP cell phenotype has not been firmly established, but the adult NP is populated with chondrocyte-like cells.^12^ Cell delivery to moderately degenerated discs has had some therapeutic potential for enhancing tissue regeneration by repopulating damaged disc tissue with cells that can restore structural and functional properties or delay degeneration. The vast majority of pre-clinical studies are performed using cells isolated from the NP without further characterization. This strategy provides a sufficient quantity of cells to conduct pre-clinical studies to ascertain benefits of delivery protocols and biomaterials. However, there are some limitations to consider in using NP cells, including limited extracellular matrix deposition, NP cell population heterogeneity, and autologous availability from patients in need. Taken together, clinical application of tissue-engineered or regenerative medicine strategies for disc repair may require alternative cell sources, such as mesenchymal stem cells (MSCs).

There are reports that the disc contains endogenous stem and progenitor cells. Risbud et al. reported that cells isolated from degenerated human tissues expressed CD105, CD166, CD63, CD49a, CD90, CD73, and CD133/1, and negative for CD34; proteins that are characteristic of marrow MSCs.^29^ When stimulated with lineage-specific induction media, these cells differentiate toward osteoblastic, chondrogenic, and adipogenic phenotypes. However, the disc pathology itself diminishes the cell's proliferation rate and differentiation potential,^97^ motivating research to identify and optimize exogenous cell populations for use in cell-based therapies for injured or degenerated discs.

Stem and progenitor cells represent an obvious candidate population for use in cell-based therapies for IVD repair. To date, most stem cells used for disc regeneration experiments are from nondisc tissues, such as bone marrow, adipose, umbilical cord blood, and synovium, due to the capacity to achieve large cell numbers from a single donor that can be used as needed (Fig. 6).^98,99^ A recent review of the literature reporting the use of MSCs in disc regeneration revealed that bone marrow-derived MSCs were the most commonly studied cell source, were largely safe and effective, and yielded superior quality of repair tissue compared with non-MSC treatments, evidenced by an increase in disc height.^100^

The shortcomings of a single cell population, such as tissue availability, dedifferentiation, or insufficient matrix production, are being addressed by cotransplantation of a primary and secondary cell population. Several studies have reported that the capacity of MSCs to differentiate and contribute to the formation of cartilaginous tissues is improved when used in conjunction with NP cells.^98,101–103^ However, improvements in tissue formation were only seen when MSCs were in direct contact with NP cells or disc tissue.^102,104^ Collectively, these studies suggest that synergy of MSCs, which are more readily available and attainable in higher numbers, together with NP cells or chondrocytes, offers a promising strategy for cell therapies for treating damaged discs. Furthermore, there is mounting evidence suggesting that disc degeneration is predominantly a function of genetics and not environmental risk factors (reviewed in Kepler et al.^105^). Therefore, it is imperative that successful treatments include a regenerative component that stimulates endogenous cells or deploys reparative cells into the disc.

### Material-based approaches

**FIG. 7.**
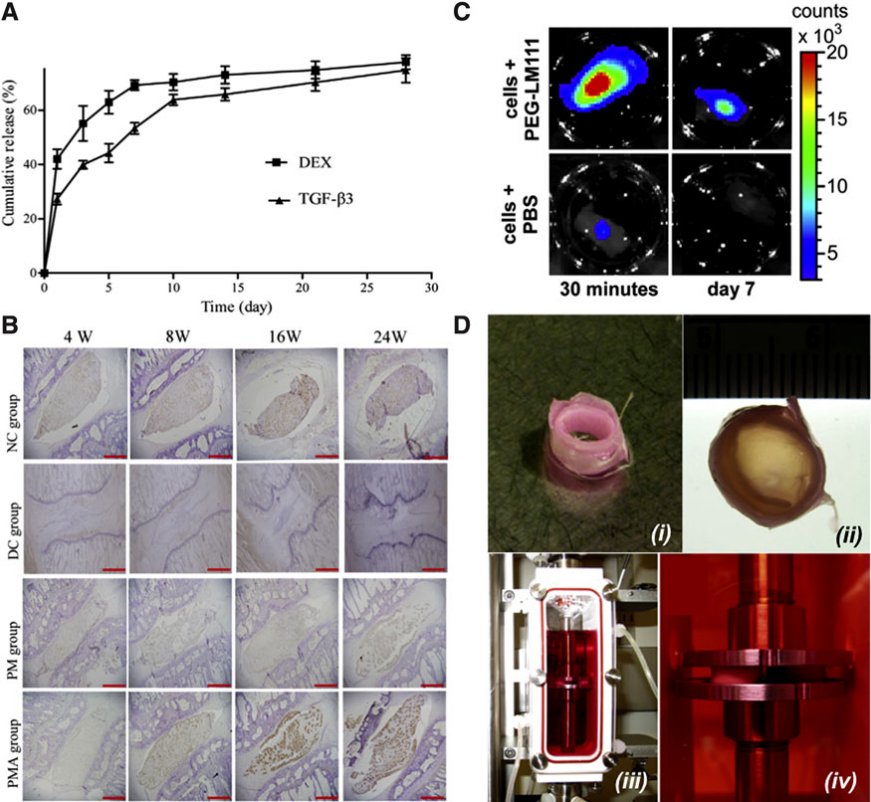
Representative tissue engineering approaches to intervertebral disc tissue engineering. **(A)** Inductive approach for treating disc degeneration through dual release of dexamethasone (DEX) and transforming growth factor-β3 (TGFβ3) from polylactic *co*-glycolic acid (PLGA) microspheres. **(B)** Immunostaining for aggregan in rodent disc samples up to 24 weeks (W) postimplantation. NC, nonoperated control; DC, degeneration control; PM, injection of DEX/TGFβ3 microspheres into disc; PMA, injection of DEX/TGFβ3 microspheres coated with adipose-derived stromal cells. **(C)** Biomaterial approach to promote survival and retention of NP cells postinjection within polyethylene glycol (PEG)–laminin-111 (LM111) biomaterial carrier (top) or phosphate-buffered saline (PBS; bottom) (30 min and 7 days postinjection). **(D)** Bioreactor-based strategy to promote the maturation of engineered discs. **(i)** Cell-laden nanofibrous strips were rolled to make a concentric ring for AF repair; **(ii)** empty core space of the concentric ring was filled with a biomaterial encapsulating human NP cells and mesenchymal stem cells to form the engineered NP; **(iii)** disc composite constructs were cultured in a bioreactor and **(iv)** stimulated by direct contact compressive loading. **(A, B)** Reprinted from Liang et al.,^108^ reprinted from Francisco et al.,^110^ and **(D)** reprinted from Tsai et al.^148^ with permission from Elsevier.

Disc replacement has emerged as the primary focus for advanced therapies in treating lower back pain associated with unmanageable pain and limited spinal motion.^106,107^ The studies reviewed here focus on NP repair or regeneration. Hydrogels possess relevant biophysical properties as a replacement material for the NP due to their ability to imbibe water, potential to withstand repeated cycles of loading, minimally invasive delivery through injection, and capacity to act as a delivery vehicle (Fig. 7).^108^

The development of an injectable biomaterial that supports cell retention, cell survival, and maintains or promotes NP phenotype *in vivo* remains a significant challenge. Injection of cells without a biomaterial commonly leads to rapid cell death or emigration from the injection site.^109–111^ In designing an injectable material to transplant cells into the disc, one must consider a number of critical parameters, including material viscosity, gelation rate, final gel stiffness, adhesivity, and degradation time. These parameters can be readily controlled by the selection of polymer composition, cross-linking method, and the incorporation of proteins or peptides that enable cell adhesion. Substrate stiffness is another key mediator of cell response that signals through regulating the intracellular cytoskeleton, activating distinct protein pathways and resultant changes in gene expression. Stiffness can be readily manipulated to achieve a targeted goal (see ND group in Table 2) by modifying the amount of polymer within the cross-linked hydrogel. Polymers derived from natural and synthetic materials are under investigation for restoring disc function.

### Natural materials

Hydrogels derived from natural polymers, including hyaluronic acid (HA), collagen, and fibrin, are the most widely used materials for NP regeneration. The fibrous morphology of these materials is comparable with native extracellular matrix. However, biomechanical properties achievable with many natural polymers are limited, constrained by available cross-linking methods and sites on the polymer backbones. Thus, there is extensive effort expended to modulate the biomechanical properties of natural materials to increase their efficacy when used as an NP implant.

HA is a major component of NP extracellular matrix that provides resistance to compression and enables cyclical loading.^112–114^ Various cell populations deployed in HA-based materials can survive and contribute to NP regeneration with no impairment in the endogenous healing processes.^115^ However, *in vivo* assessment of high-molecular-weight HA (2400–3600 kDa) alone or with cells has shown limited ability to restore disc height, which may be, in part, due to hydrogel leakage during implantation.^112,116^ These data suggest that HA has promise as a cell carrier, but the advantages may be limited without an annulus sealant to impede hydrogel extrusion from the injection site.^117,118^

Collagen is one of the most widely used materials for tissue regeneration, with numerous adhesion sites, limited immunogenicity, and injectability. Despite these advantages, collagen has not been widely used for disc repair due to poor degradation profiles and poor mechanical properties. Composite collagen hydrogels are under investigation to improve scaffold compressive mechanical properties and to control scaffold degradation rates. For example, collagen hydrogels, stabilized with a polyethylene glycol (PEG)-based cross-linker and enriched with HA, support NP cell viability *in vitro*, providing improved control over gel degradation.^119^ Others have synthesized composite collagen hydrogels supplemented with HA and proteoglycan components to improve mechanical properties, and these scaffolds have successfully maintained disc height and signal intensity on T2-weighted MRI scans.^120^

Fibrin is a naturally occurring biomaterial that provides endogenous physical and soluble cues to initiate tissue repair.^121^ Biodegradable fibrin hydrogels can be fabricated as injectable cell carriers and can be tuned by modulating clotting protein concentrations (e.g., fibrinogen and thrombin)^121^ or by altering the ionic strength of the system.^117,122^ However, fibrin-only hydrogels remain vulnerable to cell-mediated remodeling, and mechanical properties are linked to the gelation rate, which impacts survival of entrapped cells. Fibrin-HA composite hydrogels exhibit improved stability with increased glycosaminoglycan synthesis *in vitro* compared with fibrin-only gels, and NP cells deployed in this composite system demonstrated better integration with native NP tissue.^123^ The addition of silk to fibrin-HA gels significantly increased mechanical properties and enhanced chondrogenesis.^124^ Thus, the synergistic contribution of fibrin and other biomaterials represents a promising approach for use in NP repair.

### Synthetic materials

The development of synthetic materials as injectable fillers or cell carriers represents a promising strategy to combat the biomechanical limitations of natural polymer-based hydrogels. The advantages of using synthetic polymers include availability, lot-to-lot reproducibility, and elimination of contaminating biomolecules harbored within natural polymers. A number of synthetic biomaterials are under investigation for use as drug delivery vehicles and cell carriers for nuclear replacement, including PEG, polylactic *co*-glycolic acid (PLGA), polyvinyl alcohol (PVA), polyvinylpyrrolidone (PVP), and hydroxyethyl methacrylate (HEMA) (Fig. 7).^125,126^

PEG is a commonly used synthetic polymer due to its hydrophilic nature and otherwise nonfouling surface, which prevents protein adhesion. This imparts a blank slate characteristic to PEG and allows the incorporation of specific moieties that enable cell adhesion and cell-mediated degradation. Numerous chemistries are available to engineer these properties into PEG, and gelation of this polymer into a three-dimensional (3D) hydrogel can be achieved by photoinitiation or Michael-type addition reactions.^127^ Francisco et al. recently described the formation of a laminin-containing PEG hydrogel without the need for a photoinitiator in clinically relevant gelation times (10–20 min).^110^ The dynamic modulus of this hydrogel reached 1.5 kPa after gelation, which is on the same order of magnitude as previously reported values for dynamic shear modulus of human NP (7.4–19.8 kPa; Table 2).^128^ *In vivo* studies that have delivered NP cells into a degenerated disc using a PEG-laminin hydrogel showed improved initial cell retention and survival compared with an uncross-linked suspension (Fig. 7C). However, long-term cell retention was not maintained, further motivating optimization in cell delivery methods or the use of self-sealing materials to close the injection site and prevent extrusion of implanted cells.

Polyhydroxyethyl methacrylate (pHEMA) is under extensive investigation as an NP implant and can be formed to modulate mechanical properties, enable photopolymerization, and can be grafted to promote cell adhesion.^126^ Culturing pHEMA constructs under hypoxic conditions induces differentiation of MSCs toward an NP phenotype. However, matrix production of MSC-encapsulated gels was lower than the composite degradation rate, resulting in a decrease in dynamic stiffness compared with acellular gels.^126^ These studies further motivate the need for greater control over the scaffold degradation rate, which could be accomplished by adjusting the polymer concentration.

### Inductive approaches

The shortage of NP cells in affected patients is a primary driver for strategies to induce progenitor cells toward an NP phenotype. Recombinant inductive proteins are widely used to induce stem and progenitor cells toward the chondrogenic lineage. In particular, members of the transforming growth factor superfamily, such as TGFβ1, TGFβ3, and bone morphogenetic protein-2 (BMP-2), are potent chondroinductive molecules to induce MSCs toward the NP phenotype (Fig. 6).^28,129–131^ The sustained delivery of dexamethasone and TGFβ1 from PLGA microspheres has been shown to increase aggregan deposition (Fig. 7A, B). This response was enhanced when the carrier was combined with adipose-derived stromal cells, resulting in appreciable aggregan staining after 8 weeks. These data provide evidence that local presentation of inductive factors to responsive cells can stimulate matrix deposition in damaged discs.

The mitogenic potential of human NP and AF cells can be stimulated by platelet-derived growth factor, basic fibroblast growth factor, and insulin-like growth factor-1 (IGF-1).^132–135^ Delivering BMP-7 with IGF-1 synergistically stimulated proteoglycan synthesis and cell proliferation from bovine NP cells.^136^ Injecting BMP-7 alone into degenerated rabbit discs provides an initial benefit by restoring disc height.^137^ However, no beneficial effects were detected by histology, suggesting that a single injection may result in transient effects that should be further optimized.

Despite their efficacy, there are abundant concerns surrounding the clinical use of recombinant growth factors due to their short half-life, instability, increased cost, and challenges associated with binding the large molecules to polymers. Peptides can have similar efficacy while resolving the aforementioned issues with growth factors. Link-N peptide, the N-terminal part of link protein, stabilizes the link between hyaluronate and aggregan, and the local delivery of Link-N peptide promoted proteoglycan synthesis.^138^ Unfortunately, no comparison was made between Link-N and more commonly used growth factors to compare relative efficacy. Analogs for glycosaminoglycan such as pentosan polysulfate (PPS), a semisynthetic polysaccharide, can be delivered in culture media, suspended in hydrogels for local short-term stimulation,^139^ or incorporated into the backbone of hydrogels for sustained presentation.^140^ When incorporated into PEG/HA-based hydrogels, matrix deposition from MSCs was higher with bound PPS than unbound PPS.

Mechanical loading of engineered tissues for the IVD represents the fourth critical factor in a 3D culture system (Fig. 6). Dynamic loading during *de novo* tissue development improves nutrient diffusion, cell proliferation, and matrix production.^141–144^,^*^ Furthermore, loading can be used to control tissue structure development, such as collagen orientation along the direction of applied loads.^143,145,*^ For disc tissue engineering, loading may be used for the above reasons or for integrating engineered NP and AF tissues to cultivate a fully formed IVD replacement (Fig. 7D).^146–148^

### Current clinical applications for disc repair

The promising results in pre-clinical models have motivated the progression to several human clinical trials using cell-based approaches for disc degeneration. Of the three open trials (www.clinicaltrials.gov), two studies use autologous adipose stem/stromal cells (NCT02097862, NCT01643681), while the third study is testing autologous chondrocytes to promote repair (NCT01640457). Other active trials deploy allogeneic MSCs (NCT01290367, NCT01860417) to support disc height, yet the outcome of these studies has not been reported. There is no consensus on the use of a biomaterial as a carrier, although the majority of active studies employ an injectable hydrogel to deliver cells into the disc space. To date, these studies have produced mixed results with respect to increasing disc height, patient symptoms, and signal intensity on MRI, which is a measure of the tissue's water content.^149,150^ Compared with pre-clinical studies, the lack of a strong therapeutic effect suggests the need for an additional study to optimize the number of cells deployed, the use of a carrier, and the need for an annulus sealant to assist in retention of cells in the disc.

## Conclusion

Disc herniation and resultant disc degeneration remain significant clinical problems. Current effective strategies involve nonoperative management and surgical excision of the diseased fragment. Unfortunately, neither treatment addresses the underlying problem of disc injury and instability. Recent efforts to develop engineered tissues for partial or total disc repair and regenerative medicine strategies have resulted in promising outcomes *in vitro* and through small animal models. However, these strategies have significant challenges in scaling to a clinically relevant solution. Importantly, an ideal clinically relevant biological repair strategy is necessary to significantly reduce pain, restore flexibility, recapitulate the mechanical function of the healthy disc, and maintain motion of the spine.

## Abbreviations Used

AF: annulus fibrosus
BMP-2: bone morphogenetic protein-2
D: degenerate
DEX: dexamethasone
HA: hyaluronic acid
HEMA: hydroxyethyl methacrylate
IGF-1: insulin-like growth factor-1
IVD: intervertebral disc
LDH: lumbar disc herniation
MRI: magnetic resonance image
MSCs: mesenchymal stem cells
ND: nondegenerate
NP: nucleus pulposus
PBS: phosphate-buffered saline
PEG: polyethylene glycol
pHEMA: polyhydroxyethyl methacrylate
PLGA: polylactic *co*-glycolic acid
PPS: pentosan polysulfate
PVA: polyvinyl alcohol
PVP: polyvinylpyrrolidone
TGF: transforming growth factor

## Notes

^*^ O'Connell GD, Kelly TN, Roach BL, et al. Dynamic loading with chondroitinase-ABC improves collagen properties in engineered cartilage. In Review.

## References

[1] Chan WC, Sze KL, Samartzis D, et al. Structure and biology of the intervertebral disk in health and disease. Orthop Clin North Am. 2011;42:447–464 21944583 10.1016/j.ocl.2011.07.012

[2] Antoniou J, Steffen T, Nelson F, et al. The human lumbar intervertebral disc: evidence for changes in the biosynthesis and denaturation of the extracellular matrix with growth, maturation, ageing, and degeneration. J Clin Invest. 1996;98:996–1003 8770872 10.1172/JCI118884 PMC507515

[3] Beckstein JC, Sen S, Schaer TP, et al. Comparison of animal discs used in disc research to human lumbar disc: axial compression mechanics and glycosaminoglycan content. Spine (Phila Pa 1976). 2008;33:E166–E173 18344845 10.1097/BRS.0b013e318166e001

[4] Brickley-Parsons D, Glimcher MJ Is the chemistry of collagen in intervertebral discs an expression of Wolff's Law? A study of the human lumbar spine. Spine (Phila Pa 1976). 1984;9:148–163 6729579 10.1097/00007632-198403000-00005

[5] Nguyen AM, Johannessen W, Yoder JH, et al. Noninvasive quantification of human nucleus pulposus pressure with use of T1rho-weighted magnetic resonance imaging. J Bone Joint Surg Am. 2008;90:796–802 18381318 10.2106/JBJS.G.00667 PMC2657301

[6] Johnson WE, Roberts S Human intervertebral disc cell morphology and cytoskeletal composition: a preliminary study of regional variations in health and disease. J Anat. 2003;203:605–612 14686696 10.1046/j.1469-7580.2003.00249.x PMC1571197

[7] Feng G, Yang X, Shang H, et al. Multipotential differentiation of human anulus fibrosus cells: an in vitro study. J Bone Joint Surg Am. 2010;92:675–685 20194326 10.2106/JBJS.H.01672 PMC6882534

[8] Liu C, Guo Q, Li J, et al. Identification of rabbit annulus fibrosus-derived stem cells. PLoS One. 2014;9:e108239 25259600 10.1371/journal.pone.0108239 PMC4178129

[9] Henriksson H, Thornemo M, Karlsson C, et al. Identification of cell proliferation zones, progenitor cells and a potential stem cell niche in the intervertebral disc region: a study in four species. Spine (Phila Pa 1976). 2009;34:2278–2287 19755937 10.1097/BRS.0b013e3181a95ad2

[10] Eyre DR, Muir H Quantitative analysis of types I and II collagens in human intervertebral discs at various ages. Biochim Biophys Acta. 1977;492:29–42 577186 10.1016/0005-2795(77)90211-2

[11] Rodrigues-Pinto R, Richardson SM, Hoyland JA An understanding of intervertebral disc development, maturation and cell phenotype provides clues to direct cell-based tissue regeneration therapies for disc degeneration. Eur Spine J. 2014;23:1803–1814 24777668 10.1007/s00586-014-3305-z

[12] Risbud MV, Schoepflin ZR, Mwale F, et al. Defining the phenotype of young healthy nucleus pulposus cells: recommendations of the Spine Research Interest Group at the 2014 annual ORS meeting. J Orthop Res. 2015;33:283–293 25411088 10.1002/jor.22789 PMC4399824

[13] Weiler C, Nerlich AG, Schaaf R, et al. Immunohistochemical identification of notochordal markers in cells in the aging human lumbar intervertebral disc. Eur Spine J. 2010;19:1761–1770 20372940 10.1007/s00586-010-1392-z PMC2989227

[14] Melrose J, Ghosh P, Taylor TK A comparative analysis of the differential spatial and temporal distributions of the large (aggrecan, versican) and small (decorin, biglycan, fibromodulin) proteoglycans of the intervertebral disc. J Anat. 2001;198 Pt 1:3–15 11215765 10.1046/j.1469-7580.2001.19810003.x PMC1468186

[15] Roughley PJ, White RJ, Mort JS Presence of pro-forms of decorin and biglycan in human articular cartilage. Biochem J. 1996;318 Pt 3:779–784 8836119 10.1042/bj3180779 PMC1217686

[16] CDC. Prevalence and most common causes of disability among adults—United States, 2005 Available at: http://www.cdc.gov/arthritis/basics/osteoarthritis.htm (accessed 114, 2015) 19407734

[17] Kovacs FM, Arana E, Royuela A, et al. Disc degeneration and chronic low back pain: an association which becomes nonsignificant when endplate changes and disc contour are taken into account. Neuroradiology. 2014;56:25–33 24190653 10.1007/s00234-013-1294-y

[18] Atlas SJ, Keller RB, Wu YA, et al. Long-term outcomes of surgical and nonsurgical management of lumbar spinal stenosis: 8 to 10 year results from the maine lumbar spine study. Spine (Phila Pa 1976). 2005;30:936–943 15834339 10.1097/01.brs.0000158953.57966.c0

[19] Kelsey JL, Githens PB, O'Conner T, et al. Acute prolapsed lumbar intervertebral disc. An epidemiologic study with special reference to driving automobiles and cigarette smoking. Spine (Phila Pa 1976). 1984;9:608–613 6495031 10.1097/00007632-198409000-00012

[20] Miranda H, Viikari-Juntura E, Martikainen R, et al. Individual factors, occupational loading, and physical exercise as predictors of sciatic pain. Spine (Phila Pa 1976). 2002;27:1102–1109 12004179 10.1097/00007632-200205150-00017

[21] Riihimaki H, Viikari-Juntura E, Moneta G, et al. Incidence of sciatic pain among men in machine operating, dynamic physical work, and sedentary work. A three-year follow-up. Spine (Phila Pa 1976). 1994;19:138–142 8153819 10.1097/00007632-199401001-00003

[22] McCulloch JA Focus issue on lumbar disc herniation: macro- and microdiscectomy. Spine (Phila Pa 1976). 1996;21 Suppl:45S–56S 9112324 10.1097/00007632-199612151-00005

[23] Fardon DF, Milette PC, Combined Task Forces of the North American Spine Society ASoSR, et al. Nomenclature and classification of lumbar disc pathology. Recommendations of the Combined task Forces of the North American Spine Society, American Society of Spine Radiology, and American Society of Neuroradiology. Spine (Phila Pa 1976). 2001;26:E93–E113 11242399 10.1097/00007632-200103010-00006

[24] Sanchez Perez M, Gil Sierra A, Sanchez Martin A, et al. [Standardized terminology for disc disease]. Radiologia. 2012;54:503–512 22401946 10.1016/j.rx.2011.11.005

[25] Olivero WC, Wang H, Hanigan WC, et al. Cauda equina syndrome (CES) from lumbar disc herniations. J Spinal Disord Tech. 2009;22:202–206 19412023 10.1097/BSD.0b013e31817baad8

[26] Weber H Lumbar disc herniation. A controlled, prospective study with ten years of observation. Spine (Phila Pa 1976). 1983;8:131–140 6857385

[27] Saal JA, Saal JS Nonoperative treatment of herniated lumbar intervertebral disc with radiculopathy. An outcome study. Spine (Phila Pa 1976). 1989;14:431–437 2718047 10.1097/00007632-198904000-00018

[28] Abbott RD, Purmessur D, Monsey RD, et al. Regenerative potential of TGFbeta3+Dex and notochordal cell conditioned media on degenerated human intervertebral disc cells. J Orthop Res. 2012;30:482–488 21866573 10.1002/jor.21534 PMC3264846

[29] Risbud MV, Guttapalli A, Tsai TT, et al. Evidence for skeletal progenitor cells in the degenerate human intervertebral disc. Spine (Phila Pa 1976). 2007;32:2537–2544 17978651 10.1097/BRS.0b013e318158dea6

[30] Bozzao A, Gallucci M, Masciocchi C, et al. Lumbar disk herniation: MR imaging assessment of natural history in patients treated without surgery. Radiology. 1992;185:135–141 1523297 10.1148/radiology.185.1.1523297

[31] Bush K, Cowan N, Katz DE, et al. The natural history of sciatica associated with disc pathology. A prospective study with clinical and independent radiologic follow-up. Spine (Phila Pa 1976). 1992;17:1205–1212 1440010 10.1097/00007632-199210000-00013

[32] Cribb GL, Jaffray DC, Cassar-Pullicino VN Observations on the natural history of massive lumbar disc herniation. J Bone Joint Surg Br. 2007;89:782–784 17613504 10.1302/0301-620X.89B6.18712

[33] Fagerlund MK, Thelander U, Friberg S Size of lumbar disc hernias measured using computed tomography and related to sciatic symptoms. Acta Radiol. 1990;31:555–558 2278776

[34] Teplick JG, Haskin ME Spontaneous regression of herniated nucleus pulposus. AJR Am J Roentgenol. 1985;145:371–375 3875236 10.2214/ajr.145.2.371

[35] Weber H, Holme I, Amlie E The natural course of acute sciatica with nerve root symptoms in a double-blind placebo-controlled trial evaluating the effect of piroxicam. Spine (Phila Pa 1976). 1993;18:1433–1438 8235813

[36] Therapeutics I The reality of lumbar discectomy. Available at: http://in-thera.com/en/healthcare-professionals/the-reality-of-lumbar-discectomy (accessed 114, 2015)

[37] Fountas KN, Kapsalaki EZ, Feltes CH, et al. Correlation of the amount of disc removed in a lumbar microdiscectomy with long-term outcome. Spine (Phila Pa 1976). 2004;29:2521–2524; discussion 2525–2526 15543065 10.1097/01.brs.0000145413.79277.d0

[38] O'Connell GD, Malhotra NR, Vresilovic EJ, et al. The effect of discectomy and the dependence on degeneration of human intervertebral disc strain in axial compression. Spine (Phila Pa 1976). 2011;36:1765–1771 21394074 10.1097/BRS.0b013e318216752f PMC3146972

[39] Johannessen W, Auerbach JD, Wheaton AJ, et al. Assessment of human disc degeneration and proteoglycan content using T1rho-weighted magnetic resonance imaging. Spine (Phila Pa 1976). 2006;31:1253–1257 16688040 10.1097/01.brs.0000217708.54880.51 PMC2855820

[40] Osterman H, Seitsalo S, Karppinen J, et al. Effectiveness of microdiscectomy for lumbar disc herniation: a randomized controlled trial with 2 years of follow-up. Spine (Phila Pa 1976). 2006;31:2409–2414 17023847 10.1097/01.brs.0000239178.08796.52

[41] Weinstein JN, Lurie JD, Tosteson TD, et al. Surgical versus nonoperative treatment for lumbar disc herniation: four-year results for the Spine Patient Outcomes Research Trial (SPORT). Spine (Phila Pa 1976). 2008;33:2789–2800 19018250 10.1097/BRS.0b013e31818ed8f4 PMC2756172

[42] Weinstein JN, Tosteson TD, Lurie JD, et al. Surgical vs nonoperative treatment for lumbar disk herniation: the Spine Patient Outcomes Research Trial (SPORT): a randomized trial. JAMA. 2006;296:2441–2450 17119140 10.1001/jama.296.20.2441 PMC2553805

[43] O'Connell GD, Johannessen W, Vresilovic EJ, et al. Human internal disc strains in axial compression measured noninvasively using magnetic resonance imaging. Spine (Phila Pa 1976). 2007;32:2860–2868 18246009 10.1097/BRS.0b013e31815b75fb

[44] Yang KH, King AI Mechanism of facet load transmission as a hypothesis for low-back pain. Spine (Phila Pa 1976). 1984;9:557–565 6238423 10.1097/00007632-198409000-00005

[45] Cooke PM, Lutz GE Internal disc disruption and axial back pain in the athlete. Phys Med Rehabil Clin N Am. 2000;11:837–865 11092021

[46] Nachemson A The influence of spinal movements on the lumbar intradiscal pressure and on the tensil stresses in the annulus fibrosus. Acta Orthop Scand. 1963;33:183–207 14089272 10.3109/17453676308999846

[47] Bezci SE, Nandy A, O'Connell GD Effect of hydration on healthy intervertebral disk mechanical stiffness. J Biomech Eng. 2015;137; DOI: 10.1115/1.4031416 26300418

[48] van Dijk B, Potier E, Ito K Culturing bovine nucleus pulposus explants by balancing medium osmolarity. Tissue Eng Part C Methods. 2011;17:1089–1096 21718168 10.1089/ten.TEC.2011.0215

[49] O'Connell GD, Guerin HL, Elliott DM Theoretical and uniaxial experimental evaluation of human annulus fibrosus degeneration. J Biomech Eng. 2009;131:111007 20353258 10.1115/1.3212104 PMC3424515

[50] O'Connell GD, Sen S, Elliott DM Human annulus fibrosus material properties from biaxial testing and constitutive modeling are altered with degeneration. Biomech Model Mechanobiol. 2012;11:493–503 21748426 10.1007/s10237-011-0328-9 PMC3500512

[51] Adams MA, McNally DS, Dolan P ‘Stress’ distributions inside intervertebral discs. The effects of age and degeneration. J Bone Joint Surg Br. 1996;78:965–972 8951017 10.1302/0301-620x78b6.1287

[52] van Deursen LL, van Deursen DL, Snijders CJ, et al. Relationship between everyday activities and spinal shrinkage. Clin Biomech (Bristol, Avon). 2005;20:547–550 10.1016/j.clinbiomech.2005.01.005 15836943

[53] Schroeder Y, Wilson W, Huyghe JM, et al. Osmoviscoelastic finite element model of the intervertebral disc. Eur Spine J. 2006;15 Suppl 3:S361–S371 16724211 10.1007/s00586-006-0110-3 PMC2335381

[54] Cloyd JM, Malhotra NR, Weng L, et al. Material properties in unconfined compression of human nucleus pulposus, injectable hyaluronic acid-based hydrogels and tissue engineering scaffolds. Eur Spine J. 2007;16:1892–1898 17661094 10.1007/s00586-007-0443-6 PMC2223355

[55] Johannessen W, Elliott DM Effects of degeneration on the biphasic material properties of human nucleus pulposus in confined compression. Spine (Phila Pa 1976). 2005;30:E724–729 16371889 10.1097/01.brs.0000192236.92867.15

[56] Meakin JR, Hukins DW Effect of removing the nucleus pulposus on the deformation of the annulus fibrosus during compression of the intervertebral disc. J Biomech. 2000;33:575–580 10708778 10.1016/s0021-9290(99)00215-8

[57] Seroussi RE, Krag MH, Muller DL, et al. Internal deformations of intact and denucleated human lumbar discs subjected to compression, flexion, and extension loads. J Orthop Res. 1989;7:122–131 2908903 10.1002/jor.1100070117

[58] Qasim M, Natarajan RN, An HS, et al. Damage accumulation location under cyclic loading in the lumbar disc shifts from inner annulus lamellae to peripheral annulus with increasing disc degeneration. J Biomech. 2014;47:24–31 24231247 10.1016/j.jbiomech.2013.10.032

[59] Laible JP, Pflaster DS, Krag MH, et al. A poroelastic-swelling finite element model with application to the intervertebral disc. Spine (Phila Pa 1976). 1993;18:659–670 8484158 10.1097/00007632-199304000-00019

[60] Natarajan RN, Williams JR, Andersson GB Recent advances in analytical modeling of lumbar disc degeneration. Spine (Phila Pa 1976). 2004;29:2733–2741 15564922 10.1097/01.brs.0000146471.59052.e6

[61] Tsantrizos A, Ito K, Aebi M, et al. Internal strains in healthy and degenerated lumbar intervertebral discs. Spine (Phila Pa 1976). 2005;30:2129–2137 16205337 10.1097/01.brs.0000181052.56604.30

[62] Hickey DS, Hukins DW Relation between the structure of the annulus fibrosus and the function and failure of the intervertebral disc. Spine (Phila Pa 1976). 1980;5:106–116 6446156 10.1097/00007632-198003000-00004

[63] Hukins DW A simple model for the function of proteoglycans and collagen in the response to compression of the intervertebral disc. Proc Biol Sci. 1992;249:281–285 1359558 10.1098/rspb.1992.0115

[64] Kasra M, Shirazi-Adl A, Drouin G Dynamics of human lumbar intervertebral joints. Experimental and finite-element investigations. Spine (Phila Pa 1976). 1992;17:93–102 1536019 10.1097/00007632-199201000-00014

[65] O'Connell GD, Jacobs NT, Sen S, et al. Axial creep loading and unloaded recovery of the human intervertebral disc and the effect of degeneration. J Mech Behav Biomed Mater. 2011;4:933–942 21783103 10.1016/j.jmbbm.2011.02.002 PMC3143379

[66] McGlashen KM, Miller JA, Schultz AB, et al. Load displacement behavior of the human lumbo-sacral joint. J Orthop Res. 1987;5:488–496 3681523 10.1002/jor.1100050404

[67] Koeller W, Funke F, Hartmann F Biomechanical behavior of human intervertebral discs subjected to long lasting axial loading. Biorheology. 1984;21:675–686 6518283 10.3233/bir-1984-21502

[68] Markolf KL, Morris JM The structural components of the intervertebral disc. A study of their contributions to the ability of the disc to withstand compressive forces. J Bone Joint Surg Am. 1974;56:675–687 4835815

[69] Virgin WJ Experimental investigations into the physical properties of the intervertebral disc. J Bone Joint Surg Br. 1951;33-B:607–611 14880588 10.1302/0301-620X.33B4.607

[70] Bisschop A, van Dieen JH, Kingma I, et al. Torsion biomechanics of the spine following lumbar laminectomy: a human cadaver study. Eur Spine J. 2013;22:1785–1793 23460462 10.1007/s00586-013-2699-3 PMC3731493

[71] Showalter BL, Malhotra NR, Vresilovic EJ, et al. Nucleotomy reduces the effects of cyclic compressive loading with unloaded recovery on human intervertebral discs. J Biomech. 2014;47:2633–2640 24957922 10.1016/j.jbiomech.2014.05.018 PMC4164210

[72] Pfirrmann CW, Metzdorf A, Zanetti M, et al. Magnetic resonance classification of lumbar intervertebral disc degeneration. Spine (Phila Pa 1976). 2001;26:1873–1878 11568697 10.1097/00007632-200109010-00011

[73] Thompson JP, Pearce RH, Schechter MT, et al. Preliminary evaluation of a scheme for grading the gross morphology of the human intervertebral disc. Spine (Phila Pa 1976). 1990;15:411–415 2363069 10.1097/00007632-199005000-00012

[74] Alkalay RN, Burstein D, Westin CF, et al. MR diffusion is sensitive to mechanical loading in human intervertebral disks ex vivo. J Magn Reson Imaging. 2015;41:654–664 24889510 10.1002/jmri.24624 PMC9205197

[75] Cho BY, Lim J, Sim HB, et al. Biomechanical analysis of the range of motion after placement of a two-level cervical ProDisc-C versus hybrid construct. Spine (Phila Pa 1976). 2010;35:1769–1776 20395885 10.1097/BRS.0b013e3181c225fa

[76] Zirbel SA, Stolworthy DK, Howell LL, et al. Intervertebral disc degeneration alters lumbar spine segmental stiffness in all modes of loading under a compressive follower load. Spine J. 2013;13:1134–1147 23507531 10.1016/j.spinee.2013.02.010

[77] Kong MH, Morishita Y, He W, et al. Lumbar segmental mobility according to the grade of the disc, the facet joint, the muscle, and the ligament pathology by using kinetic magnetic resonance imaging. Spine (Phila Pa 1976). 2009;34:2537–2544 19841613 10.1097/BRS.0b013e3181b353ea

[78] Wu M, Wang S, Driscoll SJ, et al. Dynamic motion characteristics of the lower lumbar spine: implication to lumbar pathology and surgical treatment. Eur Spine J. 2014;23:2350–2358 24777671 10.1007/s00586-014-3316-9

[79] Jensen GM Biomechanics of the lumbar intervertebral disk: a review. Phys Ther. 1980;60:765–773 6445569 10.1093/ptj/60.6.765

[80] Fennell AJ, Jones AP, Hukins DW Migration of the nucleus pulposus within the intervertebral disc during flexion and extension of the spine. Spine (Phila Pa 1976). 1996;21:2753–2757 8979321 10.1097/00007632-199612010-00009

[81] Brault JS, Driscoll DM, Laakso LL, et al. Quantification of lumbar intradiscal deformation during flexion and extension, by mathematical analysis of magnetic resonance imaging pixel intensity profiles. Spine (Phila Pa 1976). 1997;22:2066–2072 9322316 10.1097/00007632-199709150-00002

[82] Adams MA, McNally DS, Wagstaff J, et al. Abnormal stress concentrations in lumbar intervertebral discs following damage to the vertebral bodies: a cause of disc failure? Eur Spine J. 1993;1:214–221 20054920 10.1007/BF00298362

[83] Vernon-Roberts B, Fazzalari NL, Manthey BA Pathogenesis of tears of the anulus investigated by multiple-level transaxial analysis of the T12-L1 disc. Spine (Phila Pa 1976). 1997;22:2641–2646 9399450 10.1097/00007632-199711150-00012

[84] Lawrence JP, Greene HS, Grauer JN Back pain in athletes. J Am Acad Orthop Surg. 2006;14:726–735 17148620 10.5435/00124635-200612000-00004

[85] Gunzburg R, Hutton W, Fraser R Axial rotation of the lumbar spine and the effect of flexion. An in vitro and in vivo biomechanical study. Spine (Phila Pa 1976). 1991;16:22–28 2003233 10.1097/00007632-199101000-00004

[86] Veres SP, Robertson PA, Broom ND ISSLS prize winner: how loading rate influences disc failure mechanics: a microstructural assessment of internal disruption. Spine (Phila Pa 1976). 2010;35:1897–1908 20838275 10.1097/BRS.0b013e3181d9b69e

[87] Veres SP, Robertson PA, Broom ND The influence of torsion on disc herniation when combined with flexion. Eur Spine J. 2010;19:1468–1478 20437184 10.1007/s00586-010-1383-0 PMC2989279

[88] Farfan HF, Cossette JW, Robertson GH, et al. The effects of torsion on the lumbar intervertebral joints: the role of torsion in the production of disc degeneration. J Bone Joint Surg Am. 1970;52:468–497 5425641

[89] Haughton VM, Rogers B, Meyerand ME, et al. Measuring the axial rotation of lumbar vertebrae in vivo with MR imaging. AJNR Am J Neuroradiol. 2002;23:1110–1116 12169466 PMC8185728

[90] Garges KJ, Nourbakhsh A, Morris R, et al. A comparison of the torsional stiffness of the lumbar spine in flexion and extension. J Manipulative Physiol Ther. 2008;31:563–569 18984238 10.1016/j.jmpt.2008.09.002

[91] Showalter BL, Beckstein JC, Martin JT, et al. Comparison of animal discs used in disc research to human lumbar disc: torsion mechanics and collagen content. Spine (Phila Pa 1976). 2012;37:E900–E907 22333953 10.1097/BRS.0b013e31824d911c PMC3377819

[92] Aghayev E, Etter C, Barlocher C, et al. Five-year results of lumbar disc prostheses in the SWISSspine registry. Eur Spine J. 2014;23:2114–2126 24947182 10.1007/s00586-014-3418-4

[93] Strube P, Hoff EK, Perka CF, et al. Influence of the type of the sagittal profile on clinical results of lumbar total disc replacement after a mean follow-up of 39 months. J Spinal Disord Techn. 2013 [Epub ahead of print]; DOI: 10.1097/BSD.0b013e31827f434e 23222097

[94] Yoshihara H, Yoneoka D National trends in the surgical treatment for lumbar degenerative disc disease: United States, 2000 to 2009. Spine J. 2015;15:265–271 25281920 10.1016/j.spinee.2014.09.026

[95] Nerurkar NL, Elliott DM, Mauck RL Mechanical design criteria for intervertebral disc tissue engineering. J Biomech. 2010;43:1017–1030 20080239 10.1016/j.jbiomech.2009.12.001 PMC2849875

[96] Liebscher T, Haefeli M, Wuertz K, et al. Age-related variation in cell density of human lumbar intervertebral disc. Spine (Phila Pa 1976). 2011;36:153–159 20671592 10.1097/BRS.0b013e3181cd588c

[97] Mizrahi O, Sheyn D, Tawackoli W, et al. Nucleus pulposus degeneration alters properties of resident progenitor cells. Spine J. 2013;13:803–814 23578990 10.1016/j.spinee.2013.02.065 PMC3759825

[98] Chen S, Emery SE, Pei M Coculture of synovium-derived stem cells and nucleus pulposus cells in serum-free defined medium with supplementation of transforming growth factor-beta1: a potential application of tissue-specific stem cells in disc regeneration. Spine (Phila Pa 1976). 2009;34:1272–1280 19455002 10.1097/BRS.0b013e3181a2b347

[99] Leckie SK, Sowa GA, Bechara BP, et al. Injection of human umbilical tissue-derived cells into the nucleus pulposus alters the course of intervertebral disc degeneration in vivo. Spine J. 2013;13:263–272 23384411 10.1016/j.spinee.2012.12.004 PMC4868072

[100] Yim RL, Lee JT, Bow CH, et al. A systematic review of the safety and efficacy of mesenchymal stem cells for disc degeneration: insights and future directions for regenerative therapeutics. Stem Cells Dev. 2014;23:2553–2567 25050446 10.1089/scd.2014.0203 PMC4201280

[101] Naqvi SM, Buckley CT Differential response of encapsulated nucleus pulposus and bone marrow stem cells in isolation and coculture in alginate and chitosan hydrogels. Tissue Eng Part A. 2015;21:288–299 25060596 10.1089/ten.TEA.2013.0719

[102] Richardson SM, Walker RV, Parker S, et al. Intervertebral disc cell-mediated mesenchymal stem cell differentiation. Stem Cells. 2006;24:707–716 16223853 10.1634/stemcells.2005-0205

[103] Tsaryk R, Silva-Correia J, Oliveira JM, et al. Biological performance of cell-encapsulated methacrylated gellan gum-based hydrogels for nucleus pulposus regeneration. J Tissue Eng Regen Med. 2014; DOI: 10.1002/term.1959 25370800

[104] Wei A, Chung SA, Tao H, et al. Differentiation of rodent bone marrow mesenchymal stem cells into intervertebral disc-like cells following coculture with rat disc tissue. Tissue Eng Part A. 2009;15:2581–2595 19191570 10.1089/ten.TEA.2008.0458

[105] Kepler CK, Ponnappan RK, Tannoury CA, et al. The molecular basis of intervertebral disc degeneration. Spine J. 2013;13:318–330 23537454 10.1016/j.spinee.2012.12.003

[106] Arthur A, Cannella M, Keane M, et al. Fill of the nucleus cavity affects mechanical stability in compression, bending, and torsion of a spine segment, which has undergone nucleus replacement. Spine (Phila Pa 1976). 2010;35:1128–1135 20473120 10.1097/BRS.0b013e3181bdbb1a

[107] Dahl MC, Ahrens M, Sherman JE, et al. The restoration of lumbar intervertebral disc load distribution: a comparison of three nucleus replacement technologies. Spine (Phila Pa 1976). 2010;35:1445–1453 20216342 10.1097/BRS.0b013e3181bef192

[108] Liang CZ, Li H, Tao YQ, et al. Dual release of dexamethasone and TGF-beta3 from polymeric microspheres for stem cell matrix accumulation in a rat disc degeneration model. Acta Biomater. 2013;9:9423–9433 23973308 10.1016/j.actbio.2013.08.019

[109] Bertram H, Kroeber M, Wang H, et al. Matrix-assisted cell transfer for intervertebral disc cell therapy. Biochem Biophys Res Commun. 2005;331:1185–1192 15883001 10.1016/j.bbrc.2005.04.034

[110] Francisco AT, Mancino RJ, Bowles RD, et al. Injectable laminin-functionalized hydrogel for nucleus pulposus regeneration. Biomaterials. 2013;34:7381–7388 23849345 10.1016/j.biomaterials.2013.06.038 PMC3771102

[111] Henriksson HB, Svanvik T, Jonsson M, et al. Transplantation of human mesenchymal stems cells into intervertebral discs in a xenogeneic porcine model. Spine (Phila Pa 1976). 2009;34:141–148 19112334 10.1097/BRS.0b013e31818f8c20

[112] Ghosh P, Moore R, Vernon-Roberts B, et al. Immunoselected STRO-3+ mesenchymal precursor cells and restoration of the extracellular matrix of degenerate intervertebral discs. J Neurosurg Spine (Phila Pa 1976). 2012;16:479–488 10.3171/2012.1.SPINE11852 22404141

[113] Leckie AE, Akens MK, Woodhouse KA, et al. Evaluation of thiol-modified hyaluronan and elastin-like polypeptide composite augmentation in early-stage disc degeneration: comparing 2 minimally invasive techniques. Spine (Phila Pa 1976). 2012;37:E1296–E1303 22772576 10.1097/BRS.0b013e318266ecea

[114] Malhotra NR, Han WM, Beckstein J, et al. An injectable nucleus pulposus implant restores compressive range of motion in the ovine disc. Spine (Phila Pa 1976). 2012;37:E1099–E1105 22588378 10.1097/BRS.0b013e31825cdfb7 PMC3717581

[115] Benz K, Stippich C, Fischer L, et al. Intervertebral disc cell- and hydrogel-supported and spontaneous intervertebral disc repair in nucleotomized sheep. Eur Spine J. 2012;21:1758–1768 22842955 10.1007/s00586-012-2443-4 PMC3459128

[116] Reitmaier S, Kreja L, Gruchenberg K, et al. In vivo biofunctional evaluation of hydrogels for disc regeneration. Eur Spine J. 2014;23:19–26 24121748 10.1007/s00586-013-2998-8 PMC3897837

[117] Guterl CC, Torre OM, Purmessur D, et al. Characterization of mechanics and cytocompatibility of fibrin-genipin annulus fibrosus sealant with the addition of cell adhesion molecules. Tissue Eng Part A. 2014;20:2536–2545 24684314 10.1089/ten.tea.2012.0714 PMC4161191

[118] Likhitpanichkul M, Dreischarf M, Illien-Junger S, et al. Fibrin-genipin adhesive hydrogel for annulus fibrosus repair: performance evaluation with large animal organ culture, in situ biomechanics, and in vivo degradation tests. Eur Cells Mater. 2014;28:25–37; discussion 37–38 10.22203/ecm.v028a03 PMC4409328 25036053

[119] Collin EC, Grad S, Zeugolis DI, et al. An injectable vehicle for nucleus pulposus cell-based therapy. Biomaterials. 2011;32:2862–2870 21276612 10.1016/j.biomaterials.2011.01.018

[120] Jackson AR, Huang CY, Gu WY Effect of endplate calcification and mechanical deformation on the distribution of glucose in intervertebral disc: a 3D finite element study. Comput Methods Biomech Biomed Engin. 2011;14:195–204 21337225 10.1080/10255842.2010.535815 PMC3086201

[121] Ahmed TA, Dare EV, Hincke M Fibrin: a versatile scaffold for tissue engineering applications. Tissue Eng Part B Rev. 2008;14:199–215 18544016 10.1089/ten.teb.2007.0435

[122] Davis HE, Miller SL, Case EM, et al. Supplementation of fibrin gels with sodium chloride enhances physical properties and ensuing osteogenic response. Acta Biomater. 2011;7:691–699 20837168 10.1016/j.actbio.2010.09.007

[123] Li Z, Kaplan KM, Wertzel A, et al. Biomimetic fibrin-hyaluronan hydrogels for nucleus pulposus regeneration. Regen Med. 2014;9:309–326 24935043 10.2217/rme.14.5

[124] Park SH, Cho H, Gil ES, et al. Silk-fibrin/hyaluronic acid composite gels for nucleus pulposus tissue regeneration. Tissue Eng Part A. 2011;17:2999–3009 21736446 10.1089/ten.tea.2010.0747 PMC3226034

[125] Kranenburg HJ, Meij BP, Onis D, et al. Design, synthesis, imaging, and biomechanics of a softness-gradient hydrogel nucleus pulposus prosthesis in a canine lumbar spine model. J Biomed Mater Res B Appl Biomater. 2012;100:2148–2155 22888039 10.1002/jbm.b.32780

[126] Kumar D, Gerges I, Tamplenizza M, et al. Three-dimensional hypoxic culture of human mesenchymal stem cells encapsulated in a photocurable, biodegradable polymer hydrogel: a potential injectable cellular product for nucleus pulposus regeneration. Acta Biomater. 2014;10:3463–3474 24793656 10.1016/j.actbio.2014.04.027

[127] Metters A, Hubbell J Network formation and degradation behavior of hydrogels formed by Michael-type addition reactions. Biomacromolecules. 2005;6:290–301 15638532 10.1021/bm049607o

[128] Iatridis JC, Weidenbaum M, Setton LA, et al. Is the nucleus pulposus a solid or a fluid? Mechanical behaviors of the nucleus pulposus of the human intervertebral disc. Spine (Phila Pa 1976). 1996;21:1174–1184 8727192 10.1097/00007632-199605150-00009

[129] Kim DJ, Moon SH, Kim H, et al. Bone morphogenetic protein-2 facilitates expression of chondrogenic, not osteogenic, phenotype of human intervertebral disc cells. Spine (Phila Pa 1976). 2003;28:2679–2684 14673369 10.1097/01.BRS.0000101445.46487.16

[130] Lee KI, Moon SH, Kim H, et al. Tissue engineering of the intervertebral disc with cultured nucleus pulposus cells using atelocollagen scaffold and growth factors. Spine (Phila Pa 1976). 2012;37:452–458 22037529 10.1097/BRS.0b013e31823c8603

[131] Steck E, Bertram H, Abel R, et al. Induction of intervertebral disc-like cells from adult mesenchymal stem cells. Stem Cells. 2005;23:403–411 15749935 10.1634/stemcells.2004-0107

[132] O'Connell GD, Tan AR, Palmer GD, et al. Cell migration behavior of human chondrocytes for guiding three-dimensional engineered cartilage growth. Tissue Eng Regen Med. 2015 In Press. DOI: 10.1002/term.1988

[133] O'Connell GD, Newman IB, Carapezza MA Effect of long-term osmotic loading culture on matrix synthesis from intervertebral disc cells. Biores Open Access. 2014;3:242–249 25371861 10.1089/biores.2014.0021 PMC4215332

[134] O'Connell GD, Tan AR, Cui V, et al. Human chondrocyte migration behaviour to guide the development of engineered cartilage. J Tissue Eng Regen Med. 2015; DOI: 10.1002/term.1988 PMC4531108 25627968

[135] Sampat SR, O'Connell GD, Fong JV, et al. Growth factor priming of synovium derived stem cells for cartilage tissue engineering. Tissue Eng Part A. 2011;17:2259–2265 21542714 10.1089/ten.tea.2011.0155 PMC3161099

[136] Kim JS, Ellman MB, An HS, et al. Insulin-like growth factor 1 synergizes with bone morphogenetic protein 7-mediated anabolism in bovine intervertebral disc cells. Arthritis Rheum. 2010;62:3706–3715 20812336 10.1002/art.27733 PMC2995823

[137] Imai Y, Okuma M, An HS, et al. Restoration of disc height loss by recombinant human osteogenic protein-1 injection into intervertebral discs undergoing degeneration induced by an intradiscal injection of chondroitinase ABC. Spine (Phila Pa 1976). 2007;32:1197–1205 17495776 10.1097/BRS.0b013e3180574d26

[138] Gawri R, Antoniou J, Ouellet J, et al. Best paper NASS 2013: link-N can stimulate proteoglycan synthesis in the degenerated human intervertebral discs. Eur Cells Mater. 2013;26:107–119; discussion 119 10.22203/ecm.v026a08 24027023

[139] Frith JE, Cameron AR, Menzies DJ, et al. An injectable hydrogel incorporating mesenchymal precursor cells and pentosan polysulphate for intervertebral disc regeneration. Biomaterials. 2013;34:9430–9440 24050877 10.1016/j.biomaterials.2013.08.072

[140] Frith JE, Menzies DJ, Cameron AR, et al. Effects of bound versus soluble pentosan polysulphate in PEG/HA-based hydrogels tailored for intervertebral disc regeneration. Biomaterials. 2014;35:1150–1162 24215733 10.1016/j.biomaterials.2013.10.056

[141] Albro MB, Banerjee RE, Li R, et al. Dynamic loading of immature epiphyseal cartilage pumps nutrients out of vascular canals. J Biomech. 2011;44:1654–1659 21481875 10.1016/j.jbiomech.2011.03.026 PMC3124764

[142] Albro MB, Chahine NO, Li R, et al. Dynamic loading of deformable porous media can induce active solute transport. J Biomech. 2008;41:3152–3157 18922531 10.1016/j.jbiomech.2008.08.023 PMC2633098

[143] Bian L, Fong JV, Lima EG, et al. Dynamic mechanical loading enhances functional properties of tissue-engineered cartilage using mature canine chondrocytes. Tissue Eng Part A. 2010;16:1781–1790 20028219 10.1089/ten.tea.2009.0482 PMC2952125

[144] Chahine NO, Albro MB, Lima EG, et al. Effect of dynamic loading on the transport of solutes into agarose hydrogels. Biophys J. 2009;97:968–975 19686643 10.1016/j.bpj.2009.05.047 PMC2726307

[145] Stannard JT, Edamura K, Stoker AM, et al. Development of a whole organ culture model for intervertebral disc disease. J Orthop Translat. 2015 In Press. DOI:10.1016/j.jot.2015.08.002 PMC5987001 30035069

[146] Bowles RD, Gebhard HH, Hartl R, et al. Tissue-engineered intervertebral discs produce new matrix, maintain disc height, and restore biomechanical function to the rodent spine. Proc Natl Acad Sci U S A. 2011;108:13106–13111 21808048 10.1073/pnas.1107094108 PMC3156186

[147] Martin JT, Milby AH, Chiaro JA, et al. Translation of an engineered nanofibrous disc-like angle-ply structure for intervertebral disc replacement in a small animal model. Acta Biomater. 2014;10:2473–2481 24560621 10.1016/j.actbio.2014.02.024 PMC4412172

[148] Tsai TL, Nelson BC, Anderson PA, et al. Intervertebral disc and stem cells cocultured in biomimetic extracellular matrix stimulated by cyclic compression in perfusion bioreactor. Spine J. 2014;14:2127–2140 24882152 10.1016/j.spinee.2013.11.062

[149] Yoshikawa T, Ueda Y, Miyazaki K, et al. Disc regeneration therapy using marrow mesenchymal cell transplantation: a report of two case studies. Spine (Phila Pa 1976). 2010;35:E475–E480 20421856 10.1097/BRS.0b013e3181cd2cf4

[150] Orozco L, Soler R, Morera C, et al. Intervertebral disc repair by autologous mesenchymal bone marrow cells: a pilot study. Transplantation. 2011;92:822–828 21792091 10.1097/TP.0b013e3182298a15

[151] Frei H, Oxland TR, Rathonyi GC, et al. The effect of nucleotomy on lumbar spine mechanics in compression and shear loading. Spine (Phila Pa 1976). 2001;26:2080–2089 11698883 10.1097/00007632-200110010-00007

